# Regulation of Plant Mineral Nutrition by Signal Molecules

**DOI:** 10.3390/microorganisms9040774

**Published:** 2021-04-07

**Authors:** Vipin Chandra Kalia, Chunjie Gong, Sanjay K. S. Patel, Jung-Kul Lee

**Affiliations:** 1 Department of Chemical Engineering, Konkuk University, Seoul 05029, Korea; vckaliaku@gmail.com (V.C.K.); sanjaykspatel@gmail.com (S.K.S.P.); 2 National “111” Center for Cellular Regulation and Molecular Pharmaceutics, Key Laboratory of Fermentation Engineering (Ministry of Education), Hubei University of Technology, Wuhan 430068, China; gongcj606@163.com

**Keywords:** acylhomoserine lactones, biocontrol agents, growth promoters, plant health, quorum sensing, strigolactones

## Abstract

Microbes operate their metabolic activities at a unicellular level. However, it has been revealed that a few metabolic activities only prove beneficial to microbes if operated at high cell densities. These cell density-dependent activities termed quorum sensing (QS) operate through specific chemical signals. In Gram-negative bacteria, the most widely reported QS signals are acylhomoserine lactones. In contrast, a novel QS-like system has been elucidated, regulating communication between microbes and plants through strigolactones. These systems regulate bioprocesses, which affect the health of plants, animals, and human beings. This mini-review presents recent developments in the QS and QS-like signal molecules in promoting plant health.

## 1. Introduction

Plant–microbe interactions especially in the rhizoplane, rhizosphere, endosphere, and phyllosphere form discrete ecological units, the holobionts [1,2]. Here, the different organisms are largely free living, however, quite a few develop symbiotic relationships [3,4] or pathogenic relationships, by competing for the limited available resources [5]. Two well established groups of interactions are those involving plants and (i) bacteria, and (ii) fungi [6,7,8,9,10,11]. An interesting feature of these associations is the pivotal roles played by the microbes for the development of plants by providing nutrients, vitamins, energy minerals, and protecting them from pathogens [1,2,12,13,14,15]. A few well-studied plant holobionts have focused on agriculturally important species such as grains and legumes. Microbes including bacteria, viruses, archaea, fungi, and protists are members of these ecological units. Among these microbes the most beneficial for improving plant productivity have been recognized as (i) nitrogen-fixing bacteria belonging to genera such as *Bacillus, Azotobacter*, and *Rhizobia*, and (ii) fungi belonging to phyla Glomeromycota, Basidiomycota, and Ascomycota [16,17,18,19]. Thus, for maintaining soil fertility and enhancing crop yields, QS-mediated biological processes operating in nitrogen (N) fixing bacteria and phosphorus (P) up-taking AMFs can be exploited for substituting chemical fertilizers.

**Figure 1:**
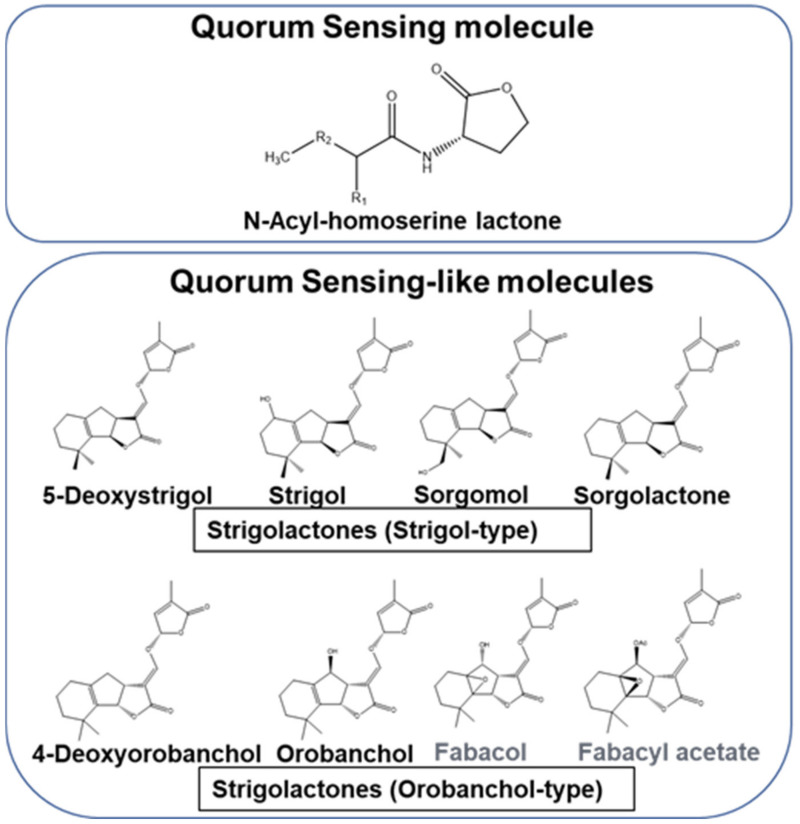
Chemical structure of quorum sensing and -like signal molecules: acylhomoserine and strigolactones. R1: H, OH or O. R2: (CH_2_)_2–14_. [22,23].

Microbial gene expression for producing biomolecules for metabolic activities and growth vary with the stage of their life cycle. It is necessary to synchronize the cell growth stage and transcription of genes for producing biomolecules in an economical and efficient manner. Certain microbes have the unique ability to express genes for specific processes only at high-cell density. This highly efficient system is termed as quorum sensing (QS) [20]. Microbes operate QS for (i) establishing a symbiotic relationship, (ii) evading competitors or stressful conditions by (a) undergoing sporulation, (b) forming biofilms, or (c) producing antimicrobial compounds [20,21]. The QS system (QSS) operation involves (a) synthesis of signal molecules, (b) their coupling to receptors, (c) binding of this complex to the promoter, and (d) gene transcription. QS signal molecules acylhomoserine lactones (AHLs) are found in Gram-negative bacteria (Figure 1), whereas in Gram-positive bacteria it is operated by autoinducing peptides, which act as autoinducers (AIs) [22,23,24]. AI-2 have been reported to act as a universal signal for QS-mediated gene expression [25]. Their primary roles are recorded in growth-promotion and protecting them from pathogens, bioremediation, and generating biofuels [26,27]. Thus, bacteria use AHLs to communicate among themselves. In contrast, Strigolactones (SLs) have been elucidated to operate as signal molecules for communication between plants and bacteria (Figure 1). It has been elucidated that SL mediated communication is a reminiscent of the bacterial QSS Their contributions in plant health and sustainability of economically important crops have been highlighted in this article.

## 2. Plant Health Regulators

Plant growth depends on the presence of macro and minor nutrients. Limitation of any one of these elements in the rhizosphere, adversely affects plant growth and results in poor yields [28]. Among the primary macronutrients, nitrogen, phosphorus, and potassium are generally the most limiting since plants require these in large amounts to carry out major metabolic activities. Commercially available fertilizers are applied to circumvent these nutrients. However, biological processes are proving to be economical and ecofriendly. Bacteria possess AHL-mediated QSS, which regulates nitrogen fixation process. On the other hand, their symbiotic partners possess SL-mediated signalling system, which promote bacterial infection and nodulation process. Similarly, plant SL sensing system sends signals to fungi to establish symbiotic association for uptake of Pi. Together these systems can help in sustainable ecosystem and higher plant growth.

### 2.1. QS Signal Molecules-AHLs

Plant growth-promoting rhizobacteria (PGPR), help plants acquire nutrients, produce growth-promoters, and protect the host from pathogenic organisms [3,4,15]. QS-mediated nitrogen fixation in *Rhizobium* promotes leguminous plant growth. QS signal–*N*-(3-oxohexadecanoyl)-L-HSL of *Sinorhizobium meliloti* 1021 enhanced the production of auxins and flavonoids to encourage the development of *Medicago truncatula* seedlings [29]. The concentration and acyl chain length of AHLs has been found to induce adventitious roots and elongation in *Arabidopsis* and mung bean plants [30,31,32,33,34,35]. *Burkholderia phytofirmans* PsJN, another PGPR, colonizes *Arabidopsis thaliana* by promoting auxin signalling [36]. Engineering the QS-mediated synthesis of the phytohormone–indole acetic acid into a non-PGPR rhizobacterium *Cupriavidus pinatubonensis* JMP134, also improved root growth rate in *A. thaliana*. QS signal molecule, *Pseudomonas* quinolone signal, mediates the acquisition of ferric iron by producing siderophores. Iron deficiency created around the plant roots prevents competing pathogens’ growth [37,38,39]. The antifungal property of *Burkholderia ambifaria* against *Rhizoctonia solani Pythium ultimum*, and *Candida albicans* is due to its ability to produce aromatic sulfur compounds and ketones [40,41]. Enacyloxin IIa produced by *B. ambifaria* AMMD^T^, inhibit the growth of the pathogen *Burkholderia multivorans* [42]. In *Pseudomonas chlororaphis* PA23, QSS-PhzI/PhzR was responsible for the enhanced production of antibiotics pyrrolnitrin (PRN) and phenazine (PHZ) and degradative enzymes such as proteases [43]. It was inhibitory against *Caenorhabditis elegans* at 0.1 µg/mL and *Sclerotinia sclerotiorum,* causing lettuce drop disease [44,45]. *P. chlororaphis* O6 present in the rhizosphere inhibited the gall producing ability of root-knot nematodes (*Meloidogyne* spp.) [46]. These “green” nematicides acted as PGPR to promote plant health [46,47,48]. QS assists plant growth by producing biomolecules that enable them to acquire nutrition and tolerate stress, making them more competitive against pathogens [49].

The role of AHLs in inducing systemic resistance against plant pathogens has a significant impact on crop yield. Expression of chitinases and salicylic acid production in the leaves protects tomatoes pathogen *Alternaria alternata* [50]. QS-regulated biocontrol agents such as *Serratia plymuthica* protects: (i) *Cucumis sativus* L. against *Botrytis cinereal*, (ii) *Phaseolus vulgaris* L., and *Lycopersicon esculentum* L. against *Pythium aphanidermatum* [51]. Treating the roots of *Hordeum vulgare* L. and *A. thaliana* with *N-*(3-oxododecanoyl)-L-HSL enabled them to develop resistance against the phytopathogens (*Blumeria graminis* and *Golovinomyces orontii*) [51]. AHLs produced by *S. meliloti* in *A. thaliana* Col-0 plants’ rhizosphere restricted the leaf infection by *Salmonella enterica* [3]. AHLs were found to cause significant changes: 3.84-fold increase in dehydroascorbate reductase activity in barley shoots and a significant decrease (23%) in superoxide dismutase activity in barley roots [4]. Exposure of excised *Arabidopsis* roots to *N*-butanoyl-L-HSL leads to a twofold increase in the intracellular Ca^2+^; an intracellular messenger causing a significant increase in the transmembrane Ca^2+^ current [52]. The expression of AHL as biocontrol agents has been attributed to the accumulation of callose and phenols, deposition of lignin, enhanced oxylipins, and salicylic acid [53,54].

### 2.2. QS Like Signal Molecules—Strigolactones

Strigolatones (SLs) are hormones which are instrumental in regulating the growth and development of plants. The release of these carotenoid derivatives into the soil act as signal molecules to communicate their presence as a host for establishing symbiotic or parasitic relationships. Here, the most prominent responders are the arbuscular mycorrhizal (AM) fungi and the opportunistic root parasitic weeds. Plants produce hormones and use them as signal molecules to communicate with other organisms and to respond to environmental stimuli [6,55,56]. SLs can also trigger germination of seeds of root parasites, i.e., *Alectra*, *Orobanche*, *Phelipanche,* and *Striga* spp. [57,58]. SLs act as chemo-attractants allowing the weed to assimilate minerals [57,59,60]. This parasitism of crops by root parasitic plants cause dramatic yield losses in legumes, cereals, rapeseed, sunflower, and tomato [61]. Although SLs promote symbiosis of roots with bacteria and fungi, parasitic weeds exploit this opportunity for their survival [6]. Plants release SLs under P deficient conditions and induce hyphal branching in AM fungi, which supports survival of both [62,63,64,65]. SLs also act as shoot branching and high-tillering inhibitors [55,56]. In fact, it has been realized that SLs regulate different aspects of plant growth and development, including tolerance of biotic and abiotic stresses [22,66,67,68,69,70,71,72,73,74,75].

#### 2.2.1. Biosynthesis and Structural Variation

Naturally occurring SLs have been extracted from various plant parts. Most of them are produced in the roots and axillary shoot buds [55,56,76]. Broadly these can be categorized into three groups. Canonical SLs have a tricycling lactone (ABC-ring) attached to a conserved lactone (butenolide, D-ring) [75]. Based on the stereochemistry of the B-/C-ring junction, these can be classified as strigol- and orobanchol- SLs. In the orobanchol-like SLs, the C-ring is in α orientation, whereas it is in β orientation in strigol-like SLs (Figure 1). In contrast, in the non-canonical SLs, the ABC-ring is replaced by different structural elements, such as epoxidation, hydroxylation, ketolation, and methylation [75,77]. A few representatives of the latter group are avenaol, heliolactone, methyl carlactonoate, and zealactone and zeapyranolactone [23]. Another group of signalling molecules related to SLs are the karrikins, which show similarity with respect to the conserved butanolide ring. These signaling molecules with a role in triggering seed germination in pioneer plants especially germinating after bush and forest fires have been perceived to participate in signal transduction pathways [78,79].

The synthesis of SLs in plants occurs through two routes: (i) mevalonate pathway, and (ii) the MEP (2-C-methyl-D-erythritol 4-phosphate) pathway. These were elucidated to take place in cytosol and plastids in maize seedlings using isoprenoid biosynthesis inhibitors: mevastatin and fosmidomycin [80]. The root exudate of maize seedlings treated with fluridone, a carotenoid biosynthesis inhibitor drastically reduced the *Striga hermonthica* seed germination [80]. Transcriptomic analysis has provided insights into the SL biosynthetic enzymes such as LATERAL BRANCHING OXIDOREDUCTASE (LBO), which in *Arabidopsis* is closely linked to MAX3 [81].

#### 2.2.2. Mechanism of Action

The mechanism of action of SLs is quite similar to that of QS molecules. However, unlike QS signal molecules, SL-mediated communication is between different organisms. This density-dependent gene regulation reflects a reminiscent of the QS signal molecules, especially AHLs, found in a diverse range of bacteria [82]. Further, to categorize SLs as “truly” QS signal molecules, a few more criteria must be met: (i) must be actively exuded by host roots into the rhizosphere [83]; (ii) act extracellularly [77]; (iii) high specificity of response (observed in plants such as *Arabidopsis*, rice, and petunia but not fully elucidated in moss) [71,84,85]; (iii) response should be at physiological concentrations (recorded in moss) [86,87,88,89,90]; (iv) beneficial to the community; (v) a direct correlation between cell density and SLs concentration (not established as yet); and (vi) response must occur above a threshold concentration (demonstrated to some extent) [86].

#### 2.2.3. Regulation of SL Production

Among the various factors responsible for plant growth and development, nutrient availability is the most critical. It has been realized that nitrogen (N) and Pi are among those nutrients, which are limiting in most soils. Plants have evolved to recruit symbiotic partners and modulate their root architecture to suit the relationship, such as nitrogen fixing bacterial nodules or AM fungi. These symbiotic partnerships help to increase N and Pi uptake [63]. In N and Pi limiting soils, biosynthesis of SLs was found to increase in red clover, rice, *Arabidopsis*, maize, and sorghum [9,91,92,93,94,95]. Effect of Pi concentration on production of heliolactone by sunflower roots, and SLs by *Physcomitrella patens* has been demonstrated using seed germination bioassays [73,96]. Beneficial effect of AM fungal (*Rhizophagus irregularis*) symbiotic relationships during nutrient deficiency conditions leading to enhanced production of SLs has been recorded in rice, *M. truncatula*, and tomato [97,98,99,100]. It may be remarked that biosynthesis of SLs, i.e., homeostasis is regulated by the presence of other plant hormones such as auxins, abscisic acid (ABA) and gibberellins (GAs) through a negative feedback mechanism [84,99,101,102,103,104,105,106].

### 2.3. Diversity of Roles of SLs

#### 2.3.1. Outgrowth of Axillary Buds

Efforts to restrict unnecessary branching in economically important plants were made by inhibiting the outgrowth of axillary buds. The evidence of the production of such a plant growth regulator in the roots and its transport to shoots was initially obtained (i) using grafting studies, where a wild-type rootstock rescued the high-tillering, and (ii) by generating plant mutants with restricted branching phenotype in *Arabidopsis*, peas, rice, and petunia [107,108,109,110].

#### 2.3.2. Regulating Branching

Non-vascular plants such as *Marchantia* (liverworts) and *Physcomitrella* (moss), exude SLs in the rhizosphere. It was observed that as a function of population density, SLs regulate colony extension and branching pattern of protonemal filaments. In fact, SLs are exuded under inorganic phosphate (Pi) limitation, leading to the optimization of uptake of Pi [9,111]. Within the plant, SLs inhibit side branching to decrease sink tissue. In addition, during the interaction with AM fungi, expansion in root surface enhances their capacity to mine Pi [86,112,113]. It helps the host to conserve energy by avoiding growth into regions depleted of Pi [86].

#### 2.3.3. Symbiotic Relationships

The role of SLs in symbiotic association between beneficial rhizobia, AMFs and the plant roots seems to be instrumental in the growth and survival of around 80% of the land plants [62,64]. These symbiotic relationships between crop plants and microbes has been widely observed. The communication between the partners through SLs as signalling molecules, enable the host to carry out diverse endogenous and exogenous activities. These signal molecules regulate seed germination, and root development, especially its architecture, density, and hair length [66,114,115,116]. Elucidation of endogenous plant hormonal roles has been done by detecting hosts by parasitic plants and AMF [6,55,56].

##### AM Fungi

Plant–microbe association in the rhizosphere, especially for Pi uptake has been described by the symbiotic AMF [67,117]. The interaction is initiated by the exudation of SLs by the roots even at extremely low concentrations of 10^−13^ M [7]. They promote AM fungal growth leading to rhizobial symbiosis. Insights into the role of SLs in AM symbiosis has been elucidated using SL-deficient plant mutants [55,83,118,119,120]. It attracts and activates hyphal branching leading to enhanced metabolism (especially Pi) leading to generation of energy and plant growth [14,121,122]. SLs (5-deoxystrigol) as an active compound as exudates in the rhizosphere of *Lotus japonicus* L. were among the first indicators of this symbiotic signalling [6]. At biochemical level, this stimulation of spore germination, hyphal growth, and cell division is linked to higher respiratory activity and biogenesis of mitochondria [7,123,124,125]. It also promotes release of short chitin oligosaccharides and cytosolic calcium uptake by the hyphae [126,127]. An interesting observation was made in *Lotus*, where induction of CLAVATA3/ESR-related (CLE) peptide in the presence of high Pi levels downregulated SL biosynthesis. It thus negatively influenced the fungal colonization in AM [128]. Under Pi limiting conditions, exposure of wheat and tomato plants to a synthetic analogue of SLs (2’-epi-GR24), promoted the accumulation of SL [129]. It also influenced the root metabolic activities, which promoted the expression of metabolites associated with Pi limitation [130]. In addition, karrikin and carlactone-type SLs have been shown to induce hyphal branching in AFM [131,132]. AMF helps plants to overcome biotic and abiotic stresses by strengthening SL production. By protecting plants from pathogens and competing with other microbes for nutrient acquisition bacteria [133,134,135].

##### Nitrogen Fixation

Despite the presence of large quantities of molecular nitrogen (N_2_) in the air, plants cannot uptake it. However, many free-living and symbiotically associated microbes especially bacteria and algae can transform N_2_ to ammonia (NH_3_) and further into nitrites, nitrates, and organic acids [136]. The plants can easily take these up to meet their N needs. Free living nitrogen fixing bacteria present in the rhizosphere includes *Azotobacter*, *Klebsiella*, *Clostridium*, and *Bacillus*. On the other hand, *Rhizobium* spp. live in symbiotic relationships with plants for operating the N fixing activities with nitrogenase enzyme help [137]. Among the economically important crops, legumes roots establish a symbiotic relationship with *Rhizobia*, which induces special structures called nodules [8,10]. The process of symbiotic nitrogen fixation is initiated by the secretion of secondary metabolites, largely flavonoids, and isoflavonoids. These molecules act as signals for activating the nodulation (Nod) genes to produce Nod proteins and factors, such as lipochito-oligosaccharides. Nodule formation and its colonization are quite specific, e.g., *S. meliloti*–*M. truncatula, Bradyrhizobium japonicum*–*Glycine max* L. Merr. (soybean) [138,139,140,141]. Many plant hormones such as cytokinins participate in developing an efficiently N fixing nodule [142,143,144].

The role of SLs in nitrogen fixation was elucidated by applying chemically synthesized SL rac-GR24 to the leguminous plants’ roots. Strong evidence of the role of SLs in stimulating nodulation in *Medicago sativa* L., alfalfa was presented by observing an enhanced expression of nod genes of *S. meliloti* strain 1021, the symbiotic nitrogen-fixing partner. GR24 at concentrations ranging from 10^−7^, 10^−5^, and 10^−3^ M was influenced the expression of *nodC* gene as seen through β-galactosidase activity. An apparent enhancement in nodules per plant supported the role of SL [145]. Further evidence on SL’s role on nodulation was recorded in *Pisum sativum* L. (pea)*-Rhizobium leguminosarum* symbiosis. A SL-deficient *rms1* mutant of pea treated with the synthetic analogue of GR24 elevated the number of nodules per g root dry weight, up from around 500 in the mutant to 900 in the treated plant [146,147]. Mutations induced in the genes responsible for SL biosynthesis in soybean and *L. japonicus*, led to drastic reduction in the number of nodules [148,149,150]. The role of SL biosynthesis and signalling was unequivocally shown in soybean (*G. max*). The expression of enzymes GmMAX1a and GmMAX4a responsible for SL biosynthesis was observed to be regulated by the infection by *Rhizobia* and their expression changed during nodule development. It also helped in increasing nodulation on soybean roots [151].

Although these studies strongly supported the role of SL in nodulation. However, there are a few exceptions, where the nodule number in the SL-insensitive ramosus4 (rms4) mutant were observed to increase [148]. Pretreatment of *M. truncatula*, with low doses of rac-GR24 (0.1 µM) stimulated nodulation. However, a negative impact was recorded at higher concentrations (2 and 5 µM). SL was influencing the early stages of nodule development and is thus critical for the symbiotic interaction [152]. The genetic makeup of the host also influences the nodulation response implying the specificity and sensitivity of SLs. Further MAX2/RMS4 along with certain other yet not identified ligands such as carotenoid-derived molecules and KARRIKIN-INSENSITIVE 2 (KAI2) are also perceived to be involved in this process [74]. In *Arabidopsis*, it has been observed that KAI2 recognizes the two stereoisomers of rac-GR24, one of them has a configuration similar to strigol, whereas the other is its enantiomer [153,154]. Correlation between SL biosynthesis and nodulation process could be elucidated through tissue-specific gene expression analysis [150,155,156,157]. rac-GR24 negatively affects the initiation of infection thread for rhizobial entry in *M. truncatula*. Pea plant carrying mutations in SL biosynthetic genes was observed to cause fewer infection threads compared to the than the parent strains [157]. This behaviour was not observed in certain mutants and in fact caused massive infection [11,152]. The Nod factor signaling pathway’s promoters were observed to be active in nodule formation and initiation of infection in *M. truncatula* nodules. Here, the role of cytokinin and auxin in increasing nodular mass has been speculated to be related to the process of autoregulation of nodulation (AON), which regulates nitrogen fixation [147,152].

#### 2.3.4. Parasitic Relationships

**Figure 2:**
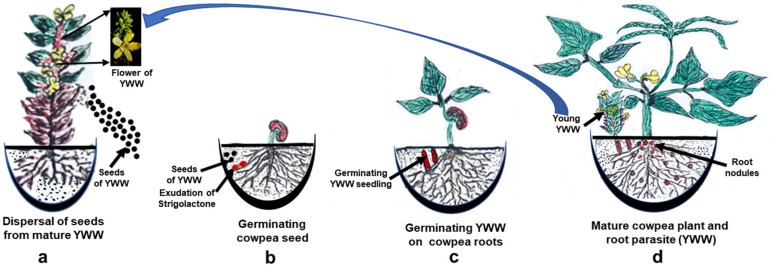
Strigolactone (SL) signal dependent induction of seed germination of root parasite *Alectra vogelii* (Yellow witch weed, YWW) on *Vigna unguiculata* L. Walp. (cowpea). (**a**): Release of large number of small sized seeds from mature YWW plant; (**b**): Exudation of SLs by germinating cowpea roots attracting YWW seeds; (**c**): Germinating YW seed (underground portion lacking photosynthetic machinery) on cowpea roots; (**d**): YWW growing as parasite along with cowpea plant. YWW plant on maturity produce a large number of seeds which have very little reserve material and have very low survival rate.

In contrast to developing symbiotic relationships, SLs also act as chemical signals, to establish a communication between weed and economically important crops (Figure 2). This relationship has a devasting effect on the crops’ productivity [158,159]. These signal molecules trigger seed germination in weeds such as *Alectra*, *Orobanche*, *Phelipanche*, and *Striga* spp. [57,58]. This synchronization between SLs and germination of seed of these parasitic plants ensure their long-term survival [160].

#### 2.3.5. Abiotic Stress

Abiotic stress conditions caused by alkalinity, salinity, drought, and temperature adversely affect yield. Chemical compounds (osmoprotectants and stimulants) such as betaine, glycine, and proline prove effective in overcoming stress and improve productivity [28]. However, rhizospheric microbes have been proving instrumental in fighting abiotic stress [161,162,163]. SLs have been proposed to stimulate resource allocation and as mediators for providing adaptive adjustments under abiotic stress conditions. Investigation of the relationship between *L. japonicus* and SLs under normal and stress conditions provided strong evidence between the two. Roots of SL-depleted plant, under P starvation and osmotic stress showed enhanced stomatal conductance. Their ability to resist drought caused by abscisic acid was also impaired due to slower stomatal closure. This proved that SLs play a significant role in drought resistance. The observation further supported that a rapid decline in SL concentration in the root exudates is recorded under osmotic stress. At the genetic level transcription of ABA biosynthetic gene *LjNCED_2_* was downregulated on pre-treatment with exogenously supplied SLs [72]. Recent work on apple plant stressed due to excessive application of potash fertilizers has been a cause of concern [164,165]. Since, SLs have been known to enable plants to tolerate NaCl and drought stresses, attempts were made to exploit them for alleviating KCl stress [166]. Exogenous spray of SLs on *Malus hupehensis* Rehd. under KCl stress revealed enhancement in the activities of catalase and peroxidase enzymes, which eliminated reactive oxygen species production. It consequently promoted, expulsion of K+ and the accumulation of proline, which thus helped in maintaining osmotic balance i.e., ion homeostasis [166]. The relationship between SLs and drought in barley (*H. vulgare*) was established using a missense mutant of the gene responsible for the expression of SL-specific receptor HvD14. Its hyper-sensitivity to stress was evident in several factors including impaired photosynthesis, altered stomatal density, and disorganized chloroplast structure. In addition, it showed major changes in the expression of genes encoding for abscisic acid and SL signalling pathway [167]. Biosynthesis of SLs under Pi starvation was observed to confer moss, *Physcomitrella patens* with a resistance against phytopathogenic fungi [73]. The role of SL as a positive regulator of leaf senescence under Pi deficiency was observed by delaying leaf senescence in *Arabidopsis oresara9* (*ore9*) mutant and *Oryza sativa* L. mutant *DWARF3* (*D3*) [168,169]. It helps reallocation of nutrients from worn out tissues to younger developing tissues. SLs thus assist plants to survive under Pi deficiency.by re-programing its adaptation strategies [170]. The need is to seek microbes which can protect plants from stress, improve yield, and protecting them against pathogens [171,172].

### 2.4. Economic Significance

The significant risk is likely to affect crops such as oilseed rape and sunflower, potentially producing biofuels. Application of naturally occurring SLs (orobanchol and 5-deoxystrigol) and their synthetic analogues (GR7, a GR24 lacking the aromatic A-ring; Nijmegen-1) can induce suicidal seed germination in weeds [173,174,175,176,177]. Naturally occurring compounds, which can mimic SLs, include dehydrocostus lactone are produced by sunflower roots. It stimulates the germination *Orobanche cumana*, a root parasite affecting sunflower [178]. Thus, encouraging AM colonization and inducing suicidal germination in parasites provide an opportunity to curtail the crop productivity’s devasting effect [179]. Although SLs stimulate root germination even at extremely low concentrations of 10 pM [179,180,181]. However, their applications though promising, are limited by high production costs, low stability, and off-target effects [158,182,183].

## 3. Other Potential QS Signals

*Burkholderia* strains are known to regulate the synthesis of antifungal compounds through CepIR QS system. *Burkholderia cenocepacia* H111 produces an unusual bioactive compound fragin, a member of diazeniumdiolate, which inhibits microbial growth and even tumors. In silico analysis revealed that genes for biosynthesis of fragin, a metal chelator are regulated by valdiazen, a new class of cell signalling molecule. It autoregulates its own biosynthesis and many other genes in a cell density-dependent manner, a characteristic of QSS [184]. Based on the homology observed among *ham* operons, it was suggested that members of the genera *Pseudomonas*, *Pandorea,* and *Burkholderia* may use the diffusible signal molecule—valdiazen (valinol diazeniumdiolate), which autoregulates its own biosynthesis and also of fragin. Fragin is a metallophore and its metal chelating ability is responsible for its antifungal property [184,185,186].

## 4. Antibiotics as QS Signals

Microbes secrete a diverse range of small molecules in their natural environments. Microbes produce antibiotics as a weapon against their predators and to attack other microbes in the environment [187]. Most studies focus on using antibiotics at concentrations that are lethal for microbial growth [188]. However, it has been questioned if these molecules ever accumulate in the environment at concentrations that may prove inhibitory to microbial growth [189]. Hence, they may have additional roles, such as participating in bacterial communication [190,191]. These antibiotics at sub-inhibitory concentrations affect QS-mediated gene expression without altering bacterial growth [192]. A few studies have revealed that antibiotics can also serve as QS signal molecules below the minimal-inhibitory concentrations (MIC) [191,193]. Using antibiotics such as rifamycin and erythromycin at low concentrations, it was possible to measure gene transcription patterns in a few promoter-*lux* reporters that were engineered into *Salmonella typhimurium* strain ATCC 14028 library. Around 5% of the QS promoters were observed to affect the functions of many *lux* genes and others involved in DNA repair, virulence, and transport [194]. Aminoglycoside antibiotics were observed to enhance bacterial biofilm formation in *Escherichia coli* and *Pseudomonas aeruginosa.* The antibiotic tobramycin was the most effective in inducing biofilm formation, whereas others such as amikacin, streptomycin, and gentamycin could induce biofilm formation to a lesser extent, 75%, 66%, and 25%, respectively [187]. Effect of antibiotics at sub-inhibitory concentrations on the expression of thirteen QS-mediated virulence-related genes was reported in *P. aeruginosa* PA01. Vancomycin was observed to enhance the transcription of *rhlAB* and *phzA2* by 10-fold. Consequently, a significant increase in the production of rhamnolipid and pyocyanin was recorded. Similar genotypic and phenotypic effects were also recorded using sub-inhibitory concentrations of ampicillin, azithromycin, and tetracycline [195]. Induction of different phenotypes was observed through the diffusion of different Rifampicin concentrations, which inhibits the transcription process, and Oligomycin A, with an ability to inhibit mitochondrial ATP synthases. The other phenotypic effects seen in *Streptomyces coelicolor* were (i) inhibition or acceleration of the development of aerial hyphae and (ii) undecylprodigiosin synthesis and inhibition of actinorhodin. These antibiotics affected QS-mediated biofilm formation at sub-inhibitory concentrations [193]. Similarly, biofilm formation in *E. coli* and *P. aeruginosa* was affected by sub-inhibitory concentrations of aminoglycoside antibiotics and β-lactam antibiotic, imipenem [187,196]. When used at concentrations like 1/4 to 1/8 of MIC (MIC of kanamycin, 8 µg/ mL), this antibiotic was observed to improve the QS-mediated violacein producing abilities of *Chromobacterium violaceum.* A few other antibiotics were also observed to show similar effects: tetracycline at 1/16 of MIC, erythromycin at 1/8 of MIC, amikacin at 1/4 of MIC and gentamycin at 1/2 of MIC [197]. Chitinase production, another QS-mediated activity, was reported to be significantly improved in the presence of antibiotics compared to control conditions. A negation of this enhanced activity in the presence of 20 µM of furanone compound 30 (C30), a known QS inhibitor, further established the role of antibiotics as QS signal [197,198]. Kanamycin at 1/6 MIC also showed a similar effect on biofilm formation [197]. Studies to elucidate the role of antibiotics at much lower concentrations using whole-transcriptome analysis of the marine bacterium *Phaeobacter inhibens* revealed an exciting role of broad-spectrum antibiotic tropodithietic acid (TDA). TDA was found to have the same regulatory functions as QS signal AHL. It was associated with LuxR-type transcriptional regulator for genes responsible for the expression of biofilm formation, motility, and antibiotic production [199]. Sub-inhibitory concentrations of natural and semisynthetic penicillins, such as oxacillin, ticarcillin, carbenicillin, and azlocillin, were shown to act as QS inducers for violacein production in *C. violaceum* strain NTCTC13274 (CV026), a mutant with an inability to synthesize AHLs [200].

## 5. Conclusions

Plant holobiont is a discrete ecological system where bacteria, fungi, and viruses interact to exchange nutrients, minerals, and produce bioactive molecules for mutual benefits. The most beneficial for improving the productivity of agricultural crops are the nitrogen-fixing bacteria and phosphate solubilizing mycorrhizal fungi. These biological processes can be most efficient if their expressions are well synchronized with the needs of the host and microbes. Here, the abilities of the plants and microbes to communicate with each other can be exploited for regulating specific gene expressions. Plants like pea, cow pea, and soybean exude signal molecules (SLs), which facilitate the entry of bacteria (*S. meliloti*)*,* into the roots of *M. truncatula* leading to nodule formation. Within the nodule these bacteria express N fixing genes under the influence of QS signals (AHLs). Similarly, SLs attract fungi (*R. irregularis*) and promote their P solubilizing activity. Thus, using leguminous plants in combination with rhizobia and fungi, it is possible to enrich soil with N and P, which in turn will aid in improving the yield of other non-leguminous crop plants specially cereals. Thus, multiple organism approach may bring us closer to “nitrogen-fixing” cereals.

## Data Availability

Not applicable.
